# Antioxidant and Nephroprotective Effects of Okra Pods Extract (*Abelmoschus esculentus* L.) against Lead Acetate-Induced Toxicity in Mice

**DOI:** 10.1155/2020/4237205

**Published:** 2020-03-25

**Authors:** Sri Puji Astuti Wahyuningsih, Nadyatul Ilma Indah Savira, Devinta Wahyu Anggraini, Dwi Winarni, Listijani Suhargo, Baskara Wiku Adi Kusuma, Faradita Nindyasari, Nur Setianingsih, Adamu Ayubu Mwendolwa

**Affiliations:** ^1^Department of Biology, Faculty of Science and Technology, Airlangga University, Surabaya 60115, Indonesia; ^2^Faculty of Medicine, Airlangga University, Surabaya 60286, Indonesia; ^3^Biology Education, Faculty of Teacher Training and Education, University of Jember, Jember, East Java 68121, Indonesia

## Abstract

In this study, we determine the curative effects of okra pods (*Abelmoschus esculentus* L.) extract against lead acetate toxicity in mice kidney. *n*-Hexane, ethyl acetate, and methanol solvent were used for extracting okra pods. The role of the extract as an antioxidant was tested by DPPH and FRAP methods. The methanol extract was used for experiments in animals. A total of 30 male BALB/c mice were randomly divided into six equal groups: normal control, negative control (lead-induced), and treatment groups (lead-induced for 28 days and administration of methanol extract at doses of 50, 100, 200, and 400 mg/kg BW for the 28 days). The following were analyzed in all groups: activity of the antioxidant enzymes, namely, superoxide dismutase (SOD) and catalase (CAT); oxidant level, namely, malondialdehyde (MDA) and nitric oxide (NO); and markers of kidney injury, namely, blood urea nitrogen (BUN) and creatinine (Cre). Kidney histopathology was also evaluated. This study showed that the methanol extract showed the highest antioxidant activity (IC_50_ is 35.21 *µ*g/mL, and FRAP is 57.58 *µ*M Fe^2+/^g). The CAT and SOD activities increased significantly in okra-treated groups (*P* < 0.05). The okra administration groups experienced a significant decrease in MDA, NO, BUN, and Cre levels (*P* < 0.05). Thickness of the epithelial proximal tubule, diameter of the proximal tubule, and percentage of necrotic cells in proximal tubule decreased, but the diameter ratio of glomerular Bowman's capsule in mice treated with okra was optimally improved and repaired like normal control (*P* < 0.05). The results of this study reveal that methanol extract has a very strong antioxidant effect and can reduce the influence of toxicity induced by lead acetate in mice kidney.

## 1. Introduction

Lead is a ubiquitous environmental toxin that has been detected in almost all phases of biological systems [1]. Lead results from the burning vehicle fuel, settles into the soil, and is absorbed by the plant, so food can be the source of lead exposure. Lead can also be found in old pipes, yellow faucets, and solder pipes that will contaminate water. Some researchers believe that lead in the water and pipeline systems results in lead poisoning [2, 3]. The kidney is one of the targeted sites of Pb-toxicity for being a major route of excretion from the body and facilitates kidney damage via oxidative stress and lipid peroxidation (LP). Acute lead poisoning (blood lead levels > 80–100 *μ*g/dL) disrupts both proximal tubular structure and function [4].

Lead may play a major role in generating oxidative stress. Several studies have reported that lead has induced oxidative stress [5]. It is believed that lead generated reactive oxygen species (ROS) and decreased the function of antioxidant enzymes; for example, it reduced glutathione (GSH) level and superoxide dismutase (SOD) activity [6, 7]. Lead acetate exposure doses of 50 mg/kg BW and 100 mg/kg BW caused oxidative stress and changed the expressions of apoptosis-related proteins in mouse liver [8]. Administration of lead acetate decreased SOD and CAT activity and increased LP in mice's kidneys [9]. The release of malondialdehyde (MDA) is an indicator of LP [10].

So far, the administration of chelating agents is generally approved for lead poisoning [11]. Natural antioxidant administration may be effective in the treatment of lead poisoning. Surprisingly, it has not been closely investigated. Some published studies have discovered that natural antioxidants have an important role in abating oxidative stress caused by lead in animals [12]. In the present study, we focused on the extract of okra pods as a natural antioxidant.

Okra, *Abelmoschus esculentus* L. is a flowering plant valued for its edible fruits and belongs to the family of Malvaceae that is often consumed as a nutritional enhancer in different countries [13]. It has been revealed that both okra fruit and seeds contain flavonoid, polyphenol, catechin, quercetin, procyanidin, and ascorbic acid functioning as antioxidants [14–16].

This study aims to investigate the antioxidant and nephroprotective potential of okra pods extract in lead acetate exposed mice. The antioxidant and nephroprotective effects of okra pods extract were measured in vitro by 2, 2-diphenyl-picrylhydrazyl (DPPH) assay and ferric reducing antioxidant power (FRAP) assay. We determined the effect of the extract in vivo by measuring the NO and MDA level, the enzyme activities of SOD and CAT, the nephroprotective effect in kidney injury (level of BUN and Cre), the thickness of epithelial proximal tubule, the diameter of the proximal tubule, the percentage of necrotic cells in the proximal tubule, and the diameter ratio of glomerular Bowman's capsule.

## 2. Materials and Methods

### 2.1. Chemicals and Reagents

*n*-Hexane and ethyl acetate were used in solvent extraction and were purchased from Fulltime Chemical (Anhui, China), and methanol was purchased from Merck (Darmstadt, Germany). DPPH was purchased from Sigma-Aldrich (MO, USA). TPTZ (2, 4, 6-tri(2-pyridyl)-s-triazine) was purchased from Oxford Laboratory (Maharashtra, India). MDA assay kit (Bioxytech® MDA-586) was purchased from Oxis International, Inc. (Portland, OR, USA). Sulfanilic acid and N-(1-naphthyl)-ethylenediamine were purchased from Merck (Darmstadt, Germany) and used for the NO assay. SOD and CAT assay kit were purchased from BioAssay Systems (Hayward, USA). BUN FS Cat. No. 1 3101 99 10 021 and Creatinine FS Cat. No. 1 1711 99 10 021 were purchased from DiaSys. For kidney histology preparations, we used neutral-buffered formalin (10%), paraffin, hematoxylin-eosin, ethanol (70%, 80%, 96%, absolute), EtOH acid, xylene, and Entellan mounting medium.

### 2.2. Plant Extract

Okra pods were collected from the traditional market in Surabaya, Indonesia, in January 2018 and identified by Dr. Junairiah, a botanist of the Department of Biology, Faculty of Science and Technology, Airlangga University. Okra pods were cleaned, sun-dried, and pulverized using a mechanical grinder. About 200 g of dried powder was extracted by reflux. In the first extraction, we used *n*-hexane (thrice, 24 h each time), and the solvents of each were collected. Ethyl acetate was used to extract the residue (thrice, 24 h each time), and the solvents of each were collected. Then the residue was extracted thrice (24 h each time) using methanol, and the solvents of each were collected. Those solvents were evaporated using a rotary evaporator and were freeze-dried. Hence, there were three extracts (*n*-hexane extract, ethyl acetate extract, and methanol extract) [17].

### 2.3. DPPH Radical Scavenging Assay

The stable DPPH free radical scavenging activity of *n*-hexane extract, ethyl acetate extract, and methanol extract was measured by Prieto's method. Each extract was prepared in various concentrations (200, 150, 100, 75, 50, 35, 25, 15, 12.5, 10, 6.25, and 3.125 *µ*g/mL). Then 100 *µ*L of each concentration was mixed with 100 *µ*L of 50 *µ*g/mL DPPH solution. After 30 min incubation, the absorbance was observed at *λ* 517 nm by UV/V is microplate spectrophotometer (Thermo Scientific™ Multiskan™ GO). DPPH, 50 *µ*g/mL, was used as DPPH absorbance and methanol as a blank. Analysis of each concentration of the extract was performed in duplicate. The percentage of radical scavenging was determined by using the following formula.

(1) $$\begin{array}{c} \%\text{DPPH scavenging}=\frac{\left(\text{Abs DPPH}-\text{Abs blank}\right)-\left(\text{Abs sample}-\text{Abs blank}\right)}{\left(\text{Abs DPPH}-\text{Abs sample}\right)}\times 100\%. \end{array}$$

Based on its calibration curve, the inhibitory concentration 50% (IC_50_) of DPPH scavenging activity of each extract could be calculated.

### 2.4. Ferric Reducing Antioxidant Power (FRAP) Assay

The principle of this method is based on the reduction of a ferric-TPTZ complex to its ferrous, colored form in the presence of antioxidants. Briefly, the FRAP reagent was prepared in acetate buffer 300 mM (pH 3.6) with 1 volume of 10 mm TPTZ solution in 40 mm hydrochloric acid and 1 volume of 20 mM ferric chloride (FeCl_3_.6H_2_O) [18]. Aliquots of 100 *µ*L sample supernatant were mixed with 900 *µ*L distilled H_2_O and 2 mL FRAP reagent. The absorbance of the reaction mixture at 593 nm was measured spectrophotometrically after incubation in the dark for 30 min. The 0.001 M ferrous sulfate (FeSO_4_·7H_2_O) was used as the standard solution. The final result was expressed as the concentration of antioxidants having a ferric reducing ability equivalent to that of 0.001 M FeSO_4_·7H_2_O (regression of standard curve using ferrous sulfate 100–2000 *µ*M). Analysis of each concentration of the extract was performed in duplicate, and the results were expressed in *µ*M Fe^2+/^g [19].

### 2.5. Animals and Experimental Design

Male BALB/c mice (8–10 weeks old, 30–40 g) were provided by the Animal Laboratory, Faculty of Pharmacy, Airlangga University, Indonesia. The animals were maintained in cages made of plastic with a lid made of woven wire cage at 20°C, with 12°h light/12°h dark cycle, fed, and watered ad libitum. All procedures involving animal care were approved by the Animal Care and Use Committee (ACUC) of Veterinary Faculty, Airlangga University, Indonesia, no. 714-KE. The mice were initially acclimatized to the experimental housing conditions and animal handlers for 7 days before all experiments to minimize handling stress during the test. Mice were randomly divided into six groups (*KN*: normal control without any treatment; *K*−: negative control induced by lead acetate 75 mg/kg BW without methanol extract administration; *P*1, *P*2, *P*3, and *P*4: methanol extract doses of 50, 100, 200, and 400 mg/kg BW, respectively). Furthermore, mice were administered lead acetate orally for 28 days (K−, *P*1, *P*2, *P*3, and *P*4). On day 29, mice were given the methanol extract orally for 28 days (*P*1, *P*2, *P*3, and *P*4). In the *KN* group, mice were given solvents only, namely, aquadest. Blood samples were collected 24 h after the last treatment. The blood was allowed to clot at room temperature and centrifuged at 3,000 rpm for 10 min to obtain serum, which was used for analyses.

### 2.6. SOD and CAT Activity Assay

The serum SOD activity was determined using EnzyChrom^TM^ Superoxide Dismutase assay kit (ESOD-100) protocols from BioAssay System (Hayward, USA). The absorbance was measured at *λ* 440 nm using a microplate UV/Vis spectrophotometer, while serum CAT activity was determined using the EnzyChrom^TM^ Catalase assay kit (ECAT-100) protocols from BioAssay System (Hayward, USA). The absorbance was measured at *λ* 570 nm using a microplate UV/Vis spectrophotometer.

### 2.7. Measurement of MDA and NO Levels

The level of MDA serum samples was analyzed using the Bioxytech® MDA-586 spectrophotometric assay kit according to the manufacturer's protocol. Briefly, the serum sample, probucol, and diluted R1 were mixed in a vortexing tube. The mixture was added to R2 and incubated at 45°C for 40 min. The tube was centrifuged (10,000 g, 10 min), and the supernatant was measured at *λ* 586 nm using a microplate UV/Vis spectrophotometer. To determine the NO concentration, the stable NO conversion product, nitrite (NO_2_), was measured with the Griess reagent [17]. For the NO assay, we used the Griess system protocol. Briefly, 50 *µ*L the serum was prepared in 1.5 mL microtube. 100 *µ*L Griess reagent I (sulfanilic acid in 2.5% phosphoric acid) and 100 *µ*L Griess reagent II (N-(1-naphthyl)-ethylenediamine in 2.5% phosphoric acid) were added to the serum. After 10–15 min incubation at room temperature, optical density values were read at 540 nm. Nitric oxide concentrations (M) were known after putting optical density values in the standard nitrite regression equation.

### 2.8. Measurement of BUN and Cre Levels

The level of BUN in serum was analyzed by Urea FS kits and Creatinine FS kits from DiaSys according to the manufacturer's protocol. The absorbance was measured using UV/Vis spectrophotometer at 340 nm for the BUN level and 492 nm for the Cre level.

### 2.9. Histopathological Study

The isolated kidney was washed using normal saline, fixed at 10% formal saline, dehydrated in ascending grades of alcohol, and embedded in paraffin wax. Sections were cut at a thickness of 4 *µ*m. Staining was done by hematoxylin and eosin (H&E). The section fields were examined under a light microscope. Furthermore, the thickness of the epithelial proximal tubule, the diameter of the proximal tubule, the percentage of necrotic cells in the proximal tubule, and diameter ratio of glomerular-Bowman's capsule were measured.

### 2.10. Statistical Analysis

Statistical data analysis was performed by one-way analysis of variance (ANOVA) followed by Duncan's post hoc test. All analyses were performed using IBM SPSS Statistics 24 software. The results were reported as the mean ± standard deviation (M ± SD) of five repeats. *P* < 0.05 was considered statistically significant.

## 3. Results and Discussion

### 3.1. Antioxidant Activity

The free radical scavenging activity of all the samples of okra pods extracts is presented in Table 1. DPPH free radicals dissolved in methanol and absorbed at *λ* 517 nm. Colors of DPPH would be changed from purple to yellow when the free radicals are scavenged by antioxidants. The methanol extract showed the highest antioxidant activity with an IC_50_ value of about 35.21 *µ*g/mL, while ethyl acetate and *n*-hexane extract showed the lowest antioxidant activity with an IC_50_ value of 181.09 and 104.06 *µ*g/mL. The FRAP assay can rank the reducing power and the antioxidant potential. The highest ability to reduce Fe^3+^ to Fe^2^+ was found in the methanol extract (57.58 *µ*M Fe^2+/^g). It means that methanol extract has stronger reducing power than *n*-hexane extract (57.13 *µ*M Fe^2+/^g) and ethyl acetate extract (49.64 *µ*M Fe^2+/^g).

The scavenging activity against DPPH free radical has been widely used to determine the antioxidant activity of plant extract [20]. Sample with IC_50_ lower than 50 *µ*g/mL can be classified as a very strong antioxidant, 50–100 *µ*g/mL as strong, 101–150 *µ*g/mL as medium, and greater than 150 *µ*g/mL as weak [21]. The results of the DPPH assay in this study showed that the methanol extract has the lowest IC_50_ value (35.21 *µ*g/mL) which indicated the best free radical scavenging activity and very strong antioxidant activity because methanol can dissolve the main active compounds such as quercetin, catechin, and vitamin, whereas *n*-hexane extract has medium antioxidant activity and ethyl acetate extract has weak antioxidant activity. DPPH values of okra pods in this study are better than those reported for okra seeds by a study from Ghana. The IC50 values for the sample infusions ranged from 127.800 to 405.667 *μ*g/ml. The undefatted samples ranged between 239.750 and 637.000 *μ*g/ml, and the defatted samples had IC50 values ranging from 28.714 to 338.333 *μ*g/ml [22].

The same thing happens when doing a FRAP test. The alcohol and ethyl acetate extracts from okra seeds contained flavonoids of 2.3328 ± 0.008 and 3.9082 ± 0.01 mg quercetin equivalent/g plant material. Both extracts are also found in rutin [23]. The ethanol extract okra seeds contain phenolics, flavonoids, total polysaccharide, and isoquercetin, while none of the four were found in *n*-hexane extract [24]. My other research showed that the total phenolic levels of okra pods in methanol extract are 12.92 mg gallic acid equivalent (GAE)/g plant material, and the flavonoid level is 5.68 mg quercetin equivalent/g plant material [25]. The methanol extract has a higher ability to reduce Fe^3+^ to Fe^2+^. Based on the DPPH and FRAP assays results, methanol extract was used to treat experimental animals with various concentrations (50, 100, 200, and 400 mg/kg BW).

This study also showed the antioxidant activity of methanol okra pods extract against lead acetate-induced toxicity in mice. In the in vivo antioxidant activity assay of methanol okra extract, we used the experimental animals to determine the kidney toxicity caused by lead acetate. Dose of exposure to lead acetate 75 mg/kg BW was chosen according to Xu et al. [8]. Parameter of toxicity could be measured from the activity of antioxidant enzymes (SOD and CAT), oxidant levels, such as NO and MDA, biochemical parameters of kidney function (BUN and Cre levels), and also histopathology of the kidney.

### 3.2. The Effect of Methanol Extract of Okra Pods on SOD and CAT Activity

**Figure 1:**
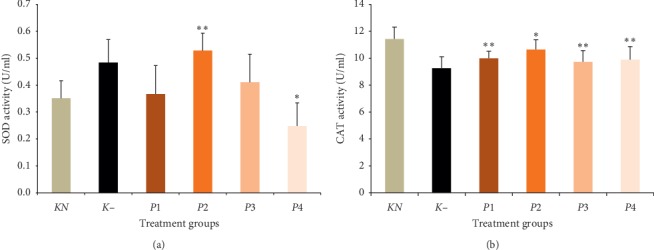
Effect of methanol extract of okra pods on both superoxide dismutase (SOD) and catalase (CAT) activity in lead acetate-induced mice. Values are as expressed as mean ± standard deviation (*n* = 5). ^*∗*^*P* < 0.05 shows significant differences in K−. ^*∗∗*^*P* < 0.05 shows significant differences in *KN*, confidence interval 95%. *KN*: normal control, *K*−: negative control. *P*1, *P*2, *P*3, and *P*4 were treated with 50, 100, 200, and 400 mg/kg BW methanol extract of okra pods, respectively.

SOD and CAT activities are presented in Figure 1. The administration of methanol extract of okra pods restores normal SOD activity, except in group *P*2, which is given methanol extract okra pods at 100 mg/kg BW. The SOD activity in *P*2 was significantly increased compared to normal and negative controls (*P* < 0.05), whereas CAT activity in negative controls was significantly lower than normal controls (*P* < 0.05). The administration of methanol extract of okra pods in groups *P*1, *P*3, and *P*4 has not been able to restore normal CAT activity (*P* > 0.05), but administration of okra extract at 100 mg/kg BW can increase and improve CAT activity as normal.

Lead could be an inhibitor of the SOD enzyme, causing reduction in the activities of both SOD [26] and CAT [27]. Contrary to expectation, this study showed that SOD activity in the lead-treated group was not increased significantly. This result was the same as that of Vaziri [20]. A possible explanation for this might be that the dose of lead was low. Furthermore, the superoxide might be the factor of enhancement of SOD activity. According to Ercal et al. [17], lead caused an increase in superoxide and other reactive oxygen species compounds. However, administration of the methanol okra extract ameliorated SOD activity, exceeding normal. The antioxidant compound of this extract might have reduced superoxide. In another result, it is reported that CAT activity was decreased in lead-treated group. This result is likely to be related to lead as an inhibitor of CAT and H_2_O_2_. Treatments of the methanol okra extract did not restore CAT activity to normal levels, but administration of 100 mg/kg BW methanol okra extract enhanced CAT activity compared to the lead-treated group. As mentioned in the literature, quercetin and other flavonoids inactivate the generation of ROS and terminate the radical chain reaction [18].

### 3.3. The Effect of Methanol Extract of Okra Pods on NO Level

**Figure 2:**
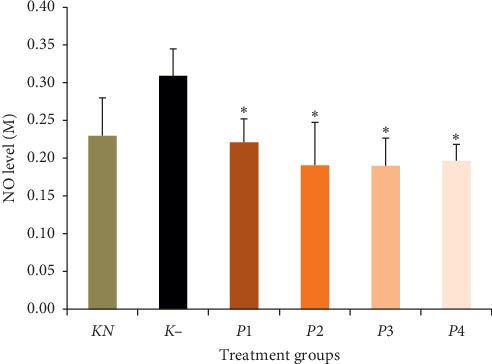
Effect of methanol extract of okra pods on nitric oxide (NO) level in lead acetate-induced mice. Values are as expressed as mean ± standard deviation (*n* = 5). ^*∗*^*P* < 0.05 shows significant differences in *K*−, confidence interval 95%. *KN*: normal control, *K*−: negative control. *P*1, *P*2, *P*3, and *P*4 were treated with 50, 100, 200, and 400 mg/kg BW methanol extract of okra pods, respectively.

The results of the experimental data on NO levels are displayed in Figure 2. Nitric oxide level was determined by nitrite (NO_2_) level, which interacted directly with sulfanilic acid under acidic conditions and was then revealed after diazotization with N-(1-naphthyl)ethylenediamine. From Figure 2, it can be seen that the NO level in the negative control (K− group) was significantly increased compared to the normal control (*P* < 0.05). The administration of methanol okra pods extracts restored NO level to normal. No significant differences were found among methanol okra pods extract treatment groups (*P* > 0.05).

Based on the results of this study, NO levels in all groups treated with okra pods were significantly lower than those in the negative control. This result was similar to those of Barbosa et al. who stated that several plants as antioxidants had strong inhibitory activity on NO production in cells [28]. In the study, nitrite concentration in plasma of lead-treated mice was higher than normal. It reflects NO synthase activity [29, 30]. Lead exposure may significantly improve endothelial NO production [8]. Nitric acid can cause damage to proteins, lipids, and deoxyribonucleic acid (DNA) either directly or after interaction with superoxide [31]. The administration of methanol extract of okra pods showed the depletion of NO level and restored it to normal.

### 3.4. The Effect of Methanol Extract of Okra Pods on MDA Level

**Figure 3:**
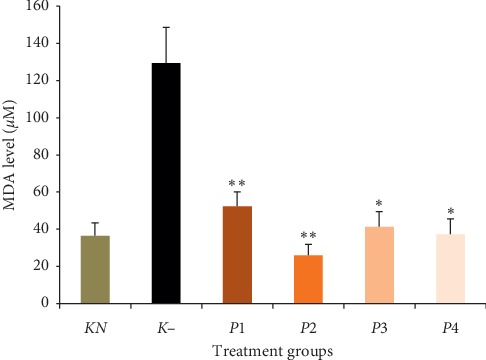
Effect of methanol extract of okra pods on malondialdehyde (MDA) level in lead acetate-induced mice. Values are as expressed as mean ± standard deviation (*n* = 5). ^*∗*^*P* < 0.05 shows significant differences with *K*−, ^*∗∗*^*P* < 0.05 shows significant differences in *KN* and K-, confidence interval 95%. *KN*: normal control, *K*−: negative control. *P*1, *P*2, *P*3, and *P*4 were treated with 50, 100, 200, and 400 mg/kg BW methanol extract of okra pods, respectively.

Figure 3 presents the results obtained from the analysis of the MDA level. Malondialdehyde as stress oxidative marker of cell shows LP. It can be seen from Figure 3 that the lead-treated group showed the highest MDA level (129.36 ± 19.2 *μ*M) and it was significantly increased by 72% compared to normal (*P* < 0.05). The MDA level of all treatments was significantly lower than the negative control (*P* < 0.05). Both *P*3 and *P*4 groups have the same MDA level as normal group (*P* > 0.05), but this is not true for both *P*1 and *P*2 groups. The *P*1 group, treated with okra methanol extract 50 mg/kg BW, were unable to return NO levels to normal. However, in group *P*2, the MDA level significantly decreased compared to the normal control group.

Lead is known to produce oxidative damage in cells by enhancing LP. Lead causes oxidative stress by stimulating the production of ROS. Reactive oxygen species attack lipids containing carbon-carbon double bond(s), especially polyunsaturated fatty acids (PUFAs) [32]. Malondialdehyde is a major reactive aldehyde resulting from LP and becoming a widely accepted biomarker [9]. This study suggested that lead administration caused a significant increase in the level of MDA serum, 72%, compared to normal. Meanwhile, the administration of methanol extract from okra pods significantly decreased MDA levels. A study by Liu et al. also stated that quercetin could decrease LP in lead-treated rat liver [33]. Quercetin is one of the antioxidant compounds in the pods extract of *A*. *esculentus* [34, 35]. Other studies have demonstrated that extract of okra fruit and seeds contains quercetin [36]. Antioxidant and metal chelating properties of quercetin depend on multiple hydroxyl groups of its chemical structure [37]. These hydroxyl groups along with the carboxyl group stabilize free radicals by donating electrons [38].

### 3.5. The Effect of Methanol Extract of Okra Pods on BUN and Creatinine Levels

**Figure 4:**
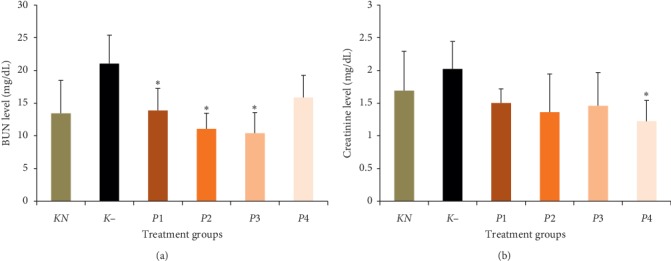
Effect of methanol extract of okra pods on blood urea nitrogen (BUN) and creatinine (Cre) levels. Values are as expressed as mean ± standard deviation (*n* = 5). ^*∗*^*P* < 0.05 shows significant differences in *K*−, confidence interval 95%. *KN*: normal control, *K*−: negative control. *P*1, *P*2, *P*3, and *P*4 were treated with 50, 100, 200, and 400 mg/kg BW methanol extract of okra pods, respectively.

The result of the experimental data on biochemical parameters in this study is measuring BUN and creatinine levels. The administration of methanol extract of okra pods significantly decreased BUN levels in *P*1, *P*2, and *P*3 groups and creatinine levels in *P*4 group (*P* < 0.05). Both BUN and Cre levels are displayed in Figure 4.

Cellular damage of kidney structure can decrease kidney function. Blood urea nitrogen and Cre levels are biochemical parameters of the kidney function. High levels of both BUN and creatinine were found in groups treated with lead acetate. This result was caused by kidney damage. This is similar to the result of Sudjarwo et al. [39] who reported that the lead acetate treatment significantly induced BUN and creatinine in serum. Our result indicated that methanol extract has a nephroprotective activity against lead acetate, as the treatment at doses of 50, 100, 200, and 400 mg/kg BW can reduce BUN and Cre levels.

### 3.6. The Effect of Methanol Extract of Okra Pods on Nephrotoxicity Kidney

**Figure 5:**
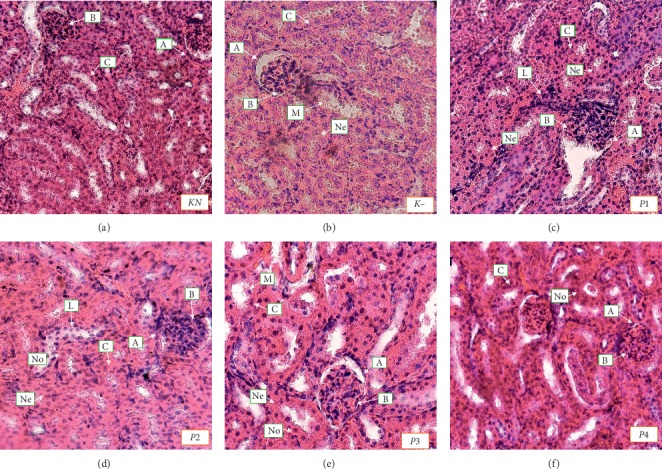
Histology of the kidney (40× objectives, scale bar 50 *μ*m). *KN*: normal control, *K*−: negative control. *P*1, *P*2, *P*3, and *P*4 were treated with 50, 100, 200, and 400 mg/kg BW methanolic extract of okra pods, respectively. A: Bowman's capsule, B: glomerular, C: proximal tubule, Ne: necrosis cell, No: normal cell, M: macrophage, L: polymorphonuclear cells.

Histopathological examination of the kidney tissue of mice that received distilled water (normal control group) showed normal nephrotic tissue. When compared with control mice (*KN*), the lead acetate-induced mice (*K*−) exhibited both swelling and necrotic cells in kidney section. Treatment with the methanol extract of okra pods (*P*1, *P*2, *P*3, and *P*4) showed kidney healing and less degree of cellular damage (Figure 5). In parallel to biochemical observation, the histopathological studies demonstrated an improvement in the kidney structure. These results are consistent with the histopathological findings of the kidney tissue from the tested mice. Furthermore, kidney tissue is further observed in the proximal tubule and the Glomerulus-Bowman capsule to measure the degree of nephrotoxicity of the kidney.

**Figure 6:**
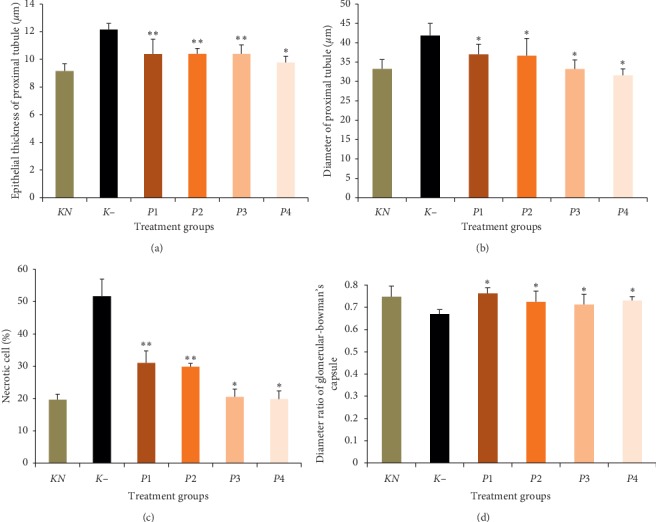
Effect of methanol extract of okra pods on kidney histology (thickness of epithelial proximal tubule, diameter of the proximal tubule, percentage of necrotic cells, and diameter ratio of glomerular Bowman's capsule) in lead acetate-treated mice. Values are as expressed as mean ± standard deviation (*n* = 5). ^*∗*^*P* < 0.05 shows significant differences in *K*−, ^*∗∗*^*P* < 0.05 shows significant differences in *KN* and *K*−, confidence interval 95%. *KN*: normal control, *K*−: negative control. *P*1, *P*2, *P*3, and *P*4 were treated with 50, 100, 200, and 400 mg/kg BW methanol extract of okra pods, respectively.

Moreover, the thickness of the epithelium, the diameter and necrotic cells of proximal tubule, and the diameter ratio of glomerular-Bowman's capsule are displayed in Figure 6. Changes in glomerular filtration greatly affect the dynamics and function of Bowman's capsules. Bowman's capsule volume has been extensively studied to maintain normal kidney function [40]. We used morphometric approaches to analyze the diameter of Bowman capsules and investigated the related factors with normal renal function. Among the cells, proximal tubules are more susceptible to Pb induced cellular damage followed by apoptosis [4]. Proximal tubule hypertrophy can be a homeostatic response to a reduced nephron number since the maximal tubular size is limited by the dimensions (luminal diameter and tubular length) that create low enough resistance to allow tubular fluid flow for adequate reabsorption [41].

It can be seen that the epithelial thickness and the diameter and percentage of necrotic cells of proximal tubule in negative control were significantly increased compared to normal control (*P* < 0.05). Furthermore, the diameter ratio of glomerular Bowman's capsule of negative control was significantly decreased (*P* < 0.05). The administration of the methanol extract of okra pods significantly decreased the thickness of the epithelial proximal tubule in the P4 group. Okra also significantly decreased the diameter of the proximal tubule and percentage of necrotic cells in both *P*3 and *P*4 groups. It also significantly increased the diameter ratio of glomerular diameter–Bowman's capsule in *P*1, *P*2, *P*3, and *P*4 groups.

Stress oxidative can cause cell damage by LP in the plasma membrane, protein modification via lead binding with sulfhydryl mediators of amino acids, and DNA mutation [42–44]. One of the potential effects of high-level lead exposure is renal injury and nephropathy. Prolonged lead exposure at lower plasma levels can cause nephrotoxicity. The most affected segment of kidney in induced renal injury is the proximal tubule [45]. Based on this result, we know that the thickness of the epithelium and the diameter and percentage of necrotic cells in proximal tubule were significantly increased, but the diameter ratio of glomerular Bowman's capsule was significantly decreased after treatment with lead acetate. Rana et al. reported that Pb hinders the integrity of the cell junctions (tight junction) and modifies cellular structure. Altered polarity and vectorial transport of epithelial cells could result from atypical cell-cell junction structure following reduced renal proximal tubule lumen and microvilli loss [4].

The epithelial thickness and the diameter of the proximal tubule are directly proportional. Enhancement of thickness of the epithelium and the diameter of the proximal tubule is caused by cell swelling, which is a change in cell environment or a way of cell adaptation to repair damage. Microscopically cell injury results in cell swelling, sometimes followed by compression or displacement position of cell organelle [46]. Percentage of necrotic cells indicated that cells have been damaged. The percentage of necrotic cells increased significantly after lead acetate-induced toxicity. Other that than, extract methanol of okra pods can repair the damage of these cells by their antioxidant activity. Based on this result, treatment of methanol extract can decrease cell damage, and then there is a decrease in the epithelial thickness and the diameter and percentage of necrotic cells in the proximal tubule effectively at dose 50 mg/kg BW and optimally at doses 200 and 400 mg/kg BW. Kotyk et al. reported that the glomerular diameter increased in fructose-supplemented rats when compared to controls [47]. Furthermore, the effect of methanol extract of okra pods can improve the immune system, which is at dose of 100 mg/kg BW could increase NK cell activity and IFN-*γ* levels [48]. Thus, many immune cells like macrophages and neutrophils increase to repair and eliminate cell debris. 200 and 400 mg/kg BW are doses for repair toward normal.

## 4. Conclusions

We concluded that the methanol extract of okra pods can attenuate lead toxicity by increasing levels of SOD and CAT and decreasing levels of MDA, NO, BUN, and Cre. Okra pods succeeded in stimulating the histology of kidney like thickness of the epithelial proximal tubule, diameter of the proximal tubule, percentage of necrotic cells, and ratio diameter of glomerular Bowman's capsule toward the normal. Okra has nephroprotective potential against the toxicity of the acetate lead in mice. This suggests that the methanol extract of okra pods has antioxidant activity and plays a protective role against lead-induced toxicity.

## Figures and Tables

**Table 1:** Result of antioxidant activity from DPPH and FRAP assays of various okra pods extracts.

Sample extract	IC_50_ DPPH assay (*µ*g/mL)	FRAP assay (*µ*M Fe^2+/^g)
*n*-Hexane extract	104.06	57.13
Ethyl acetate extract	181.09	49.64
Methanolic extract	35.21	57.58

## Data Availability

All data arising from this study are included within the article.

## Abbreviations

DPPH: 2, 2-Diphenyl-picrylhydrazyl
FRAP: Ferric reducing antioxidant power
SOD: Superoxide dismutase
CAT: Catalase
MDA: Malondialdehyde
NO: Nitric oxide
BUN: Blood urea nitrogen
Cre: Creatinine.
